# Chemical Analysis of Cod Liver Oil

**Published:** 1849-07

**Authors:** John C. Baker

**Affiliations:** No. 100 North Third street


### *Chemical Analysis of Cod Liver Oil.*

[As the internal administration of this article as a therapeutic agent in the treatment of scrofula and tuberculosis, is at the present time attracting much attention, the following carefully prepared comparative analysis of the different varieties of the oil, by a highly respectable druggist of this city, may not prove unacceptable to our readers. By these it will be seen that the light brown oil contains the largest proportions of the supposed active ingredient—*Iodine.*—Eds. Exam.]

Philadelphia, June 20th, 1849.

Editors of the Medical Examiner:

*Dear Sirs:*—As it is of the utmost importance that the cod liver oil should be chemically pure, and free from admixture of vegetable or animal oil, to meet the indications for which it is prescribed by the physician, we have obtained careful analyses of the oil, and examined a number of specimens, and have arrived at the conclusion, that the light brown oil is that which contains the largest proportion of the active constituents. It is thought by many physicians that the white cod liver oil is the most efficient, but upon submitting our analyses and samples to a few scientific medical gentlemen of this city, they have pronounced the light brown oil to be more efficient than any other in use.

To prove the correctness of the foregoing statement, and to afford to every member of the medical profession an opportunity of judging for himself, we annex the analyses to which reference is made above. Yours truly,

John C. Baker,
No. 100 North Third street

*Comparative Analysis of the three different kinds of Cod-Liver Oil.*

| | Brown. | Light Brown. | Light. |
|---|---|---|---|
| Oleic acid, (with Godwin,) - - | 69.78500 | 71.75700 | 74.03300 |
| Margaric acid, - - - - - | 16.44500 | 15.42100 | 11.75700 |
| Glycerine, - - - - - - | 9.71100 | 9.07300 | 10.17700 |
| Butter acid, - - - - - | 0.15875 | | 0.07436 |
| Acetic acid, - - - - - | 0.12506 | | 0.04571 |
| Fellic or cholic acid, with traces of margerin, alein and bilifulvin, | 0.29900 | 0.06200 | 0.04300 |
| Bilifulvin, Billifellic acid, and two other peculiar substances, - | 0.87600 | 0.44500 | 0.26800 |
| A peculiar substance soluble in alcohol of 30°, - - - - | 0.03800 | 0.01300 | 0.00600 |
| A peculiar substance insoluble in water, alcohol and ether, - | 0.00500 | 0.00200 | 0.00100 |
| Iodine, - - - - - - | 0.02950 | 0.04060 | 0.03740 |
| Chlorine and traces of bromine, - | 0.08400 | 0.15880 | 0.14880 |
| Phosphoric acid, - - - - | 0.05365 | 0.07890 | 0.09133 |
| Sulphuric acid, - - - - - | 0.01010 | 0.08595 | 0.07100 |
| Phosphorus, - - - - - | 0.00754 | 0.01136 | 0.02125 |
| Lime, - - - - - - - | 0.08170 | 0.16780 | 0.15150 |
| Magnesia, - - - - - - | 0.00380 | 0.01230 | 0.00886 |
| Soda, - - - - - - | 0.01790 | 0.06810 | 0.05540 |
| Iron, - - - - - - | Traces. | | |
| Loss, - - - - - - - | 2.56900 | 2.60319 | 3.00943 |
| | 100.00000 | 100.00000 | 100.00000 |

